# Population-based trends and underlying risk factors for infant respiratory syncytial virus and bronchiolitis hospitalizations

**DOI:** 10.1371/journal.pone.0205399

**Published:** 2018-10-31

**Authors:** Mihoko V. Bennett, Kimmie McLaurin, Christopher Ambrose, Henry C. Lee

**Affiliations:** 1 Division of Neonatal & Developmental Medicine, Stanford School of Medicine, Stanford, California, United States of America; 2 California Perinatal Quality Care Collaborative, Stanford, California, United States of America; 3 AstraZeneca, Gaithersburg, Maryland, United States of America; Louisiana State University System, UNITED STATES

## Abstract

**Objective:**

Respiratory syncytial virus (RSV) is a common pathogen during infancy, with the potential to cause serious disease and mortality in high-risk groups. The objective of this study was to characterize trends of RSV and bronchiolitis hospitalizations in the first year in a population-based cohort and assess differences in trends according to risk status.

**Methods:**

Using an observational retrospective cohort design, we examined a California population-based dataset of vital statistics linked to hospital discharge data for up to 1 year after birth from 1997–2011. Infants were categorized by medical condition and then by gestational age. Medical conditions of interest included chronic lung disease, certain congenital heart diseases, or others known to affect risk for developing severe bronchiolitis. The primary outcome was hospitalization due to RSV; secondary outcome was hospitalization for unspecified bronchiolitis (UB) not coded as RSV. Annual person-year rates were calculated for infants <12 months of age during January to December of each year.

**Results:**

Of 7,298,401 infants born during the study period, 121,230 (1.7%) had a medical condition associated with risk; these infants experienced 6853 RSV and 6568 UB hospitalizations in the first year. In infants without medical conditions, 96,694 RSV and 69,886 UB hospitalizations occurred. All-cause infant hospitalizations declined over time from 12.2 to 9.3 per 100 person-years. RSV hospitalization rates for infants with medical conditions decreased from 7.6 to 3.4 per 100 person-years, with the largest relative decline in infants with chronic lung disease (12.0 to 5.0 per 100 person-years). For infants without medical conditions, RSV hospitalizations declined from 1.4 to 0.8 per 100 person-years, with greater decreases among preterm infants with earlier gestational age. UB hospitalization rates remained relatively stable across the study years, from 6.2 to 5.4 and 1.0 to 0.8 per 100 person-years for infants with and without medical conditions.

**Conclusions:**

Various interventions may have contributed to observed decreases in RSV hospitalizations from 1998–2011, which were greater in high-risk populations recommended for RSV immunoprophylaxis and not observed with UB. Further efforts to promote evidence-based practice and optimal targeting of appropriate interventions will ensure continued improvement in care for vulnerable infants.

## Introduction

Respiratory syncytial virus (RSV) is a leading cause of hospitalization and can result in serious morbidity and mortality in infants, particularly infants born preterm or with a high-risk medical condition such as chronic lung disease (CLD) or congenital heart disease (CHD) [1–4]. Because of the high burden of disease, interventions to prevent serious RSV disease have been implemented in the past 2 decades for these high-risk infants [5–7]. There have been reports of reduced incidence of RSV and/or bronchiolitis hospitalization in recent years for infants overall and specifically for infants with CLD and CHD [1, 8, 9]. However, none of these studies have employed a cohort design in which the population is prospectively followed forward in time; instead, they have used estimated denominator populations based on national birth statistics. Furthermore, due to this design limitation, no study has examined hospitalization rates among preterm infants because gestational age (GA) at birth was not available for hospitalized infants. It is not clear whether observations of reduced hospitalizations for RSV and bronchiolitis may be due to improvements and changes in the care of neonatal and infant respiratory disease in general, or whether prevention and intervention measures targeted directly at RSV have been particularly effective. Specifically, RSV immunoprophylaxis of high-risk infants with palivizumab was increasingly utilized in the United States during the study period following its licensure in 1998.

In this study, we assessed infant hospitalization trends for RSV and unspecified bronchiolitis (UB) by presence of higher-risk medical conditions and GA during the first year of life in a population-based cohort. We compared trends for those infants to those without higher-risk medical conditions. By using statewide California databases linked with patient identifiers, we tracked infants from birth through the first year of life and accounted for admissions to any hospital in the state.

## Methods

California hospital discharge data from the Office of Statewide Hospital Planning & Development (OSHPD) linked to vital statistics for 1997–2011 were used to create a longitudinal, population-based cohort of newborns followed to 1 year of age. These years were those for which complete data were available. The OSHPD database of hospital discharges captures almost all hospital births and subsequent hospital admissions in California, with the exception of military hospitals or birthing centers not reporting to OSHPD (<3% of all deliveries). Although there is a separate record for each hospitalization, a unique identifier for each individual allows matching of patients across their first year after delivery. The probabilistic linkage algorithm used to perform the patient match by OSHPD is robust and described in detail previously [10]. In addition to various sociodemographic characteristics, each record contains a list of International Classification of Diseases-9 (ICD-9) codes to characterize the admission.

Birth hospitalizations were identified using ICD-9 codes V30.XX–V39.XX as in previous work and included delivery hospitalizations as well as subsequent transferred admissions [1]. California birth hospitalizations regardless of state of residence were included; the large majority (>98%) were California residents. We included infants who had GA of 22–44 weeks recorded on their birth certificates. Individuals with potential errors in coding GA, death during delivery or during the birth hospitalization, or inconsistent data, such as admission occurring after the date of death, were excluded.

Infants were categorized according to medical condition as noted in S1 Table. Any diagnosis occurring at any time point during the first year of life was assigned as a characteristic of that infant. CHD diagnoses were characterized into high-risk or low-risk strata consistent with previous research [1], with high-risk superseding low-risk status. As a category for analysis, Down syndrome without CHD was considered separately and was mutually exclusive of higher-risk or lower-risk CHD. A category of “other high-risk” was created, encompassing cystic fibrosis with pulmonary manifestations, neuromuscular disease, human immunodeficiency virus, immunodeficiency, and congenital and metabolic disease. The other high-risk group excluded infants with CLD, high-risk or low-risk CHD, Down syndrome, and congenital anomalies of the respiratory system.

For the analyses of risk among infants by GA without other serious medical conditions, infants with CLD, congenital anomalies of the respiratory system, higher-risk CHD, or the other high-risk conditions were excluded. Down syndrome and lower-risk CHD were not excluded for this analysis, as these conditions have not traditionally been targeted for RSV-specific prevention efforts. A sensitivity analysis excluding lower-risk CHD and Down syndrome was performed to compare the results in infants without any comorbid conditions.

The outcomes of interest were hospital admission related to either RSV or UB occurring prior to 12 months of age. We identified transfers based on discharge from one hospital and admission to another hospital on the same date; transfers were classified as a single hospital admission. Patients with any relevant ICD-9 codes during the hospitalization for RSV (480.1, 466.11, 079.6) or UB (466.19, 466.1) listed as a diagnostic code were identified, with those having both considered as having RSV.

Annual trends of RSV- and UB-coded nonbirth hospital admissions during the first year after birth were compared by medical condition and then for those without medical conditions by GA category (22–28, 29–31, 32–34, 35–37, and 38–44 completed weeks GA). When presenting results for trends over time with years as the unit, the birth year of the patient was the basis for categorization. For example, if a nonbirth hospitalization for an infant born in November 2011 occurred in January 2012, this would be categorized as a 2011 patient and hospitalization. Infants were followed for 12 months or until death if this occurred within the first year of life. During follow-up, all hospitalizations, including repeat hospitalizations, were considered to be the event. Hospitalization rates were estimated per 100 person-years and were assessed for trends over time using unadjusted Poisson regression. By evaluating person-time incidence, we were able to adjust the exposure denominator for infants who died and were no longer eligible to be hospitalized, as well as adjust the numerator for infants who may have been hospitalized more than once for RSV or UB.

Analyses were conducted using SAS 9.4 software (SAS Institute, Cary, NC). This study was approved by the Stanford University Institutional Review Board. The patient data used in this study were fully anonymized prior to our access, and the Institutional Review Board waived the requirement for informed consent.

## Results

**Fig 1:**
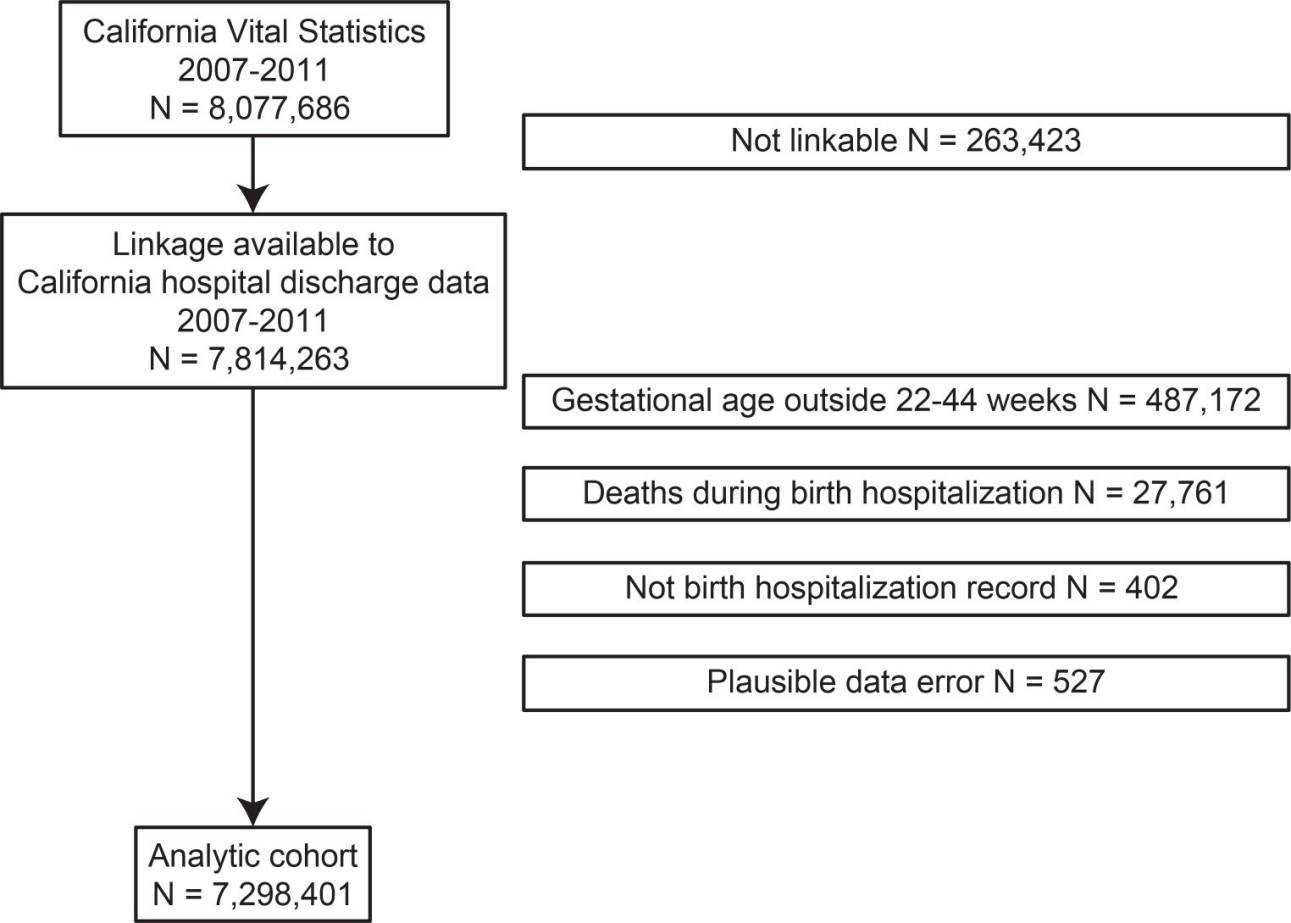
Patient cohort.

After exclusions, 7,298,401 infants were born and discharged to their homes after the birth hospitalization during the study period (Fig 1). In this cohort, 121,230 infants (1.7%) had an identified medical condition (Table 1). In this cohort of infants with medical conditions, 95,785 nonbirth hospitalizations occurred during the first year of life; there were 6853 (7.2%) hospitalizations for RSV and 6568 (6.9%) for UB. The proportion of infants hospitalized for RSV and UB were 5.4% (6580 infants) and 4.9% (5915 infants), respectively in this cohort. Among infants without identified medical conditions, 712,135 nonbirth hospitalizations occurred: 96,694 (13.6%) for RSV and 69,886 (9.8%) for UB. The proportion of infants hospitalized was 1.3% (95,270 infants) for RSV and 0.9% (66,891 infants) for UB. Although RSV and UB were more common reasons for hospitalization in the cohort without medical conditions, hospitalization rates were higher for infants with medical conditions (5.9 per 100 person-years for both RSV and UB) compared with those without (1.3 and 1.0 per 100 person-years for RSV and UB, respectively). All-cause infant hospitalization declined by 24%, from 12.2 per 100 person-years in 1998 to 9.3 per 100 person-years in 2011 (ptrend <0.001).

**Table 1:** Nonbirth hospitalizations by medical condition.

Medical Condition	Number of Infants	Number of Hospitalizations During the First Year	Number of RSV Hospitalizations During the First Year	Number of UB Hospitalizations
Infants with identified medical conditions				
Chronic respiratory disease arising in the perinatal period (ie, chronic lung disease)	19,347	13,905	1316	1651
Congenital anomalies of the respiratory system	18,900	20,076	1207	1646
Higher-risk congenital heart disease	63,893	49,775	3760	3134
Lower-risk congenital heart disease	91,944	30,547	3324	2987
Down syndrome without congenital heart disease	4463	2220	293	276
Cystic fibrosis with pulmonary manifestations	845	1142	73	71
Neuromuscular disease	2553	4568	261	269
Human immunodeficiency virus	3	3	0	0
Immunodeficiency	1923	3372	204	208
Congenital and metabolic	32,325	29,862	1519	1500
Infants without serious medical conditions by GA				
22–28 weeks	24,963	3607	546	478
29–31 weeks	52,690	6637	1076	920
32–34 weeks	194,821	21,829	3733	2558
35–37 weeks	1,062,530	112,944	16,787	11,693
≥38 weeks	5,842,167	487,074	73,128	51,242

GA, gestational age; RSV, respiratory syncytial virus; UB, unspecified bronchiolitis.

**Fig 2:**
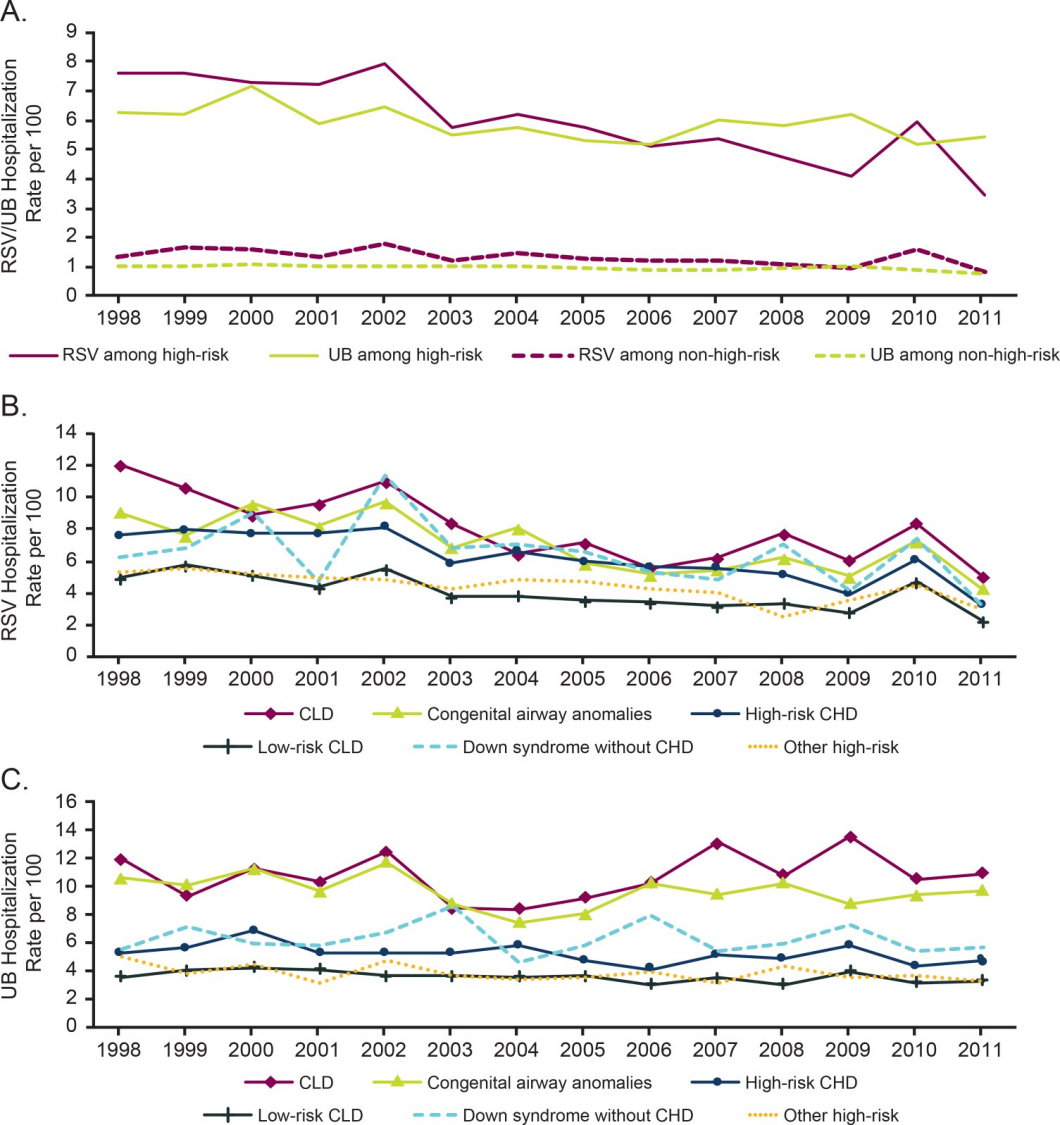
Rates by identified medical condition. (A) Rates of RSV and UB by identified medical condition status. (B) Rates of RSV by identified medical condition. (C) Rates of UB by identified medical condition. CHD, congenital heart disease; CLD, chronic lung disease; RSV, respiratory syncytial virus; UB, unspecified bronchiolitis.

For infants without identified medical conditions, the rate of RSV hospitalization ranged from 0.8 to 1.8 hospitalizations per 100 person-years across birth year cohorts (Fig 2A). The rate was 1.4 hospitalizations per 100 person-years in 1998, 1.6 per 100 person-years in 2010, and 0.8 hospitalizations per 100 person-years in 2011 (ptrend <0.001). The rate of UB hospitalization was less variable, with annual rates ranging from 0.8 to 1.1 hospitalizations per 100 person-years across the study period (ptrend <0.001). For infants with identified medical conditions, RSV hospitalizations decreased across the study period, from 7.6 per 100 person-years in 1998 to 3.4 per 100 person-years in 2011 (Fig 2A) (ptrend <0.001).

RSV hospitalizations decreased significantly for all high-risk conditions (Fig 2B). The largest relative drop occurred in infants with CLD (from 12.0 to 5.0 per 100 person-years; ptrend <0.001) and high-risk CHD (7.6 to 3.2 per 100 person-years; ptrend <0.001). Decreased rates were also seen for infants with congenital airway anomalies (9.1 to 4.3 per 100 person-years; ptrend <0.001), low-risk CHD (4.9 to 2.2 per 100 person-years; ptrend = 0.001), Down syndrome without CHD (6.2 to 3.2 per 100 person-years; ptrend = 0.026), and other high-risk conditions (5.3 to 3.0 per 100 person-years; ptrend <0.001).

To assess changes associated with years in which RSV immunoprophylaxis was increasingly used among high-risk populations [11, 12], trend tests were performed separately for 1998–2004 and 2005–2011. RSV hospitalization rates for infants with CLD decreased during 1998–2004 (ptrend <0.001); however, the hospitalization rate did not decrease significantly after 2005. The RSV hospitalization rate for congenital airway anomalies declined over time (ptrend <0.001), although the decreasing trends during 1998–2004 and 2005–2011 were not statistically significant during each time period. The trend of RSV hospitalization rates for high-risk CHD was examined from 1998–2002 and 2003–2011, as RSV immunoprophylaxis was first approved for this population in 2003. Although the rates of high-risk CHD were not significantly different between 1998 and 2002 (ptrend = 0.6347), rates did decrease significantly after 2003 (ptrend <0.001). The rates of UB hospitalizations among infants with identified medical conditions showed less change (Fig 2C).

**Fig 3:**
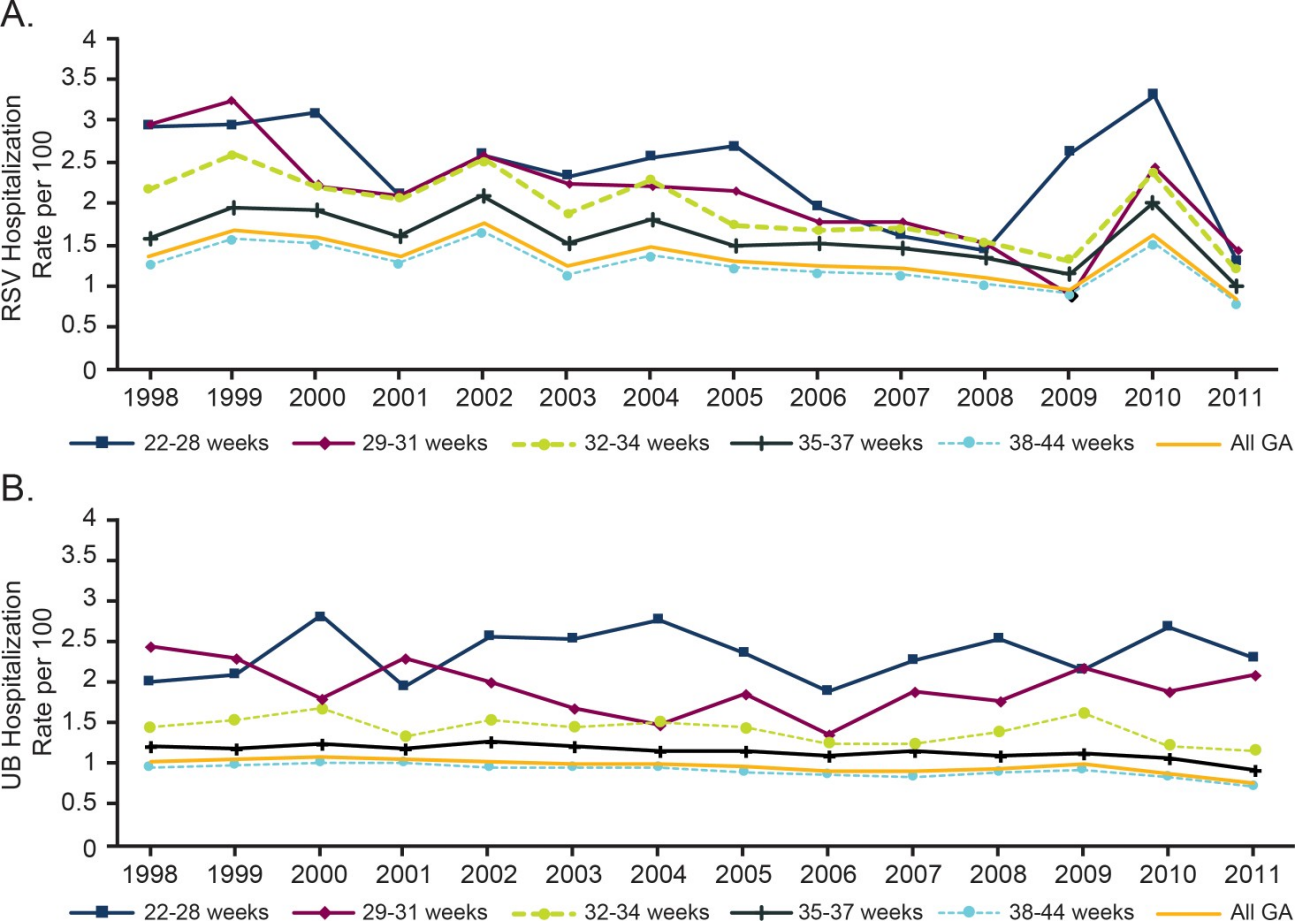
Rates of hospitalization by GA in infants without medical conditions. (A) Rates of RSV by GA in infants without a medical condition. (B) Rates of UB by GA in infants without medical a condition. GA, gestational age; RSV, respiratory syncytial virus; UB, unspecified bronchiolitis.

For infants without medical conditions, rates of RSV hospitalizations were highest for the most preterm infants and decreased with increasing GA (Fig 3A). RSV hospitalization rates decreased for all preterm GA categories, with greater decreases in those with earlier GA: 22–28 weeks (from 2.9 to 1.3 per 100 person-years), 29–31 weeks (from 3.0 to 1.4 per 100 person-years), 32–34 weeks (from 2.2 to 1.2 per 100 person-years), 35–37 weeks (from 1.6 to 1.0 per 100 person-years), and 38–44 weeks (from 1.3 to 0.8 per 100 person-years). Given the relative population sizes, the largest number of hospitalizations occurred in the 38–44 weeks GA group (n = 74,176) compared with all other GA groups combined (n = 22,518). The sensitivity analysis excluding all comorbid conditions showed similar results.

Rates of UB remained more stable across GA groups (Fig 3B). A similar pattern of decreased risk by older GA was seen for UB hospitalizations. The total number of UB hospitalizations was 53,475 for infants 38–44 weeks GA and 16,411 for infants 22–37 weeks GA. RSV represented a lower proportion of all bronchiolitis (RSV or UB) hospitalizations among more preterm infants.

RSV and UB hospitalizations by sociodemographic and clinical characteristics are shown in Table 2. Privately insured patients were less likely to be hospitalized in general, but particularly so for RSV and UB. This was true for infants with and without identified medical conditions.

**Table 2:** Risk for RSV and UB hospitalization by high-risk and low-risk status.

		High Risk for RSV								Non–High Risk for RSV							
		Total Infants		Infants With Readmissions		Infants With Nonbirth RSV		Infants With Nonbirth UB		Total Infants		Infants With Readmissions		Infants With Nonbirth RSV		Infants With Nonbirth UB	
		N = 121,230		N = 59,945		Hospitalizations		Hospitalizations		N = 7,177,171		N = 632,091		Hospitalizations		Hospitalizations	
						N = 6580		N = 5915						N = 95,270		N = 66,891	
		Count	%	Count	%	Count	%	Count	%	Count	%	Count	%	Count	%	Count	%
Gestational age, wk	22–28	13,945	11.5	4338	7.2	519	7.9	685	11.6	24,963	0.3	3607	0.6	546	0.6	478	0.7
	29–31	7257	6.0	2210	3.7	317	4.8	329	5.6	52,690	0.7	6637	1.1	1076	1.1	920	1.4
	32–34	9017	7.4	3421	5.7	466	7.1	403	6.8	194,821	2.7	21,829	3.5	3733	3.9	2558	3.8
	35–37	20,413	16.8	10,580	17.6	1179	17.9	971	16.4	1,062,530	14.8	112,944	17.9	16,787	17.6	11,693	17.5
	38–44	70,598	58.2	39,396	65.7	4099	62.3	3527	59.6	5,842,167	81.4	487,074	77.1	73,128	76.8	51,242	76.6
Birth weight, g	<750	5305	4.4	1754	2.9	196	3.0	265	4.5	2830	0.0	519	0.1	44	0.0	62	0.1
	750–999	6706	5.5	2010	3.4	249	3.8	311	5.3	7205	0.1	1312	0.2	162	0.2	198	0.3
	1000–1249	5237	4.3	1582	2.6	225	3.4	263	4.4	12,778	0.2	1940	0.3	288	0.3	288	0.4
	1250–1499	3622	3.0	1061	1.8	151	2.3	159	2.7	19,912	0.3	2643	0.4	426	0.4	352	0.5
	1500–2499	17,368	14.3	7220	12.0	888	13.5	759	12.8	373,382	5.2	40,521	6.4	6787	7.1	4616	6.9
	≥2500	82,991	68.5	46,318	77.3	4871	74.0	4158	70.3	6,761,051	94.2	585,155	92.6	87,563	91.9	61,375	91.8
Sex	Female	56,816	46.9	26,607	44.4	2839	43.2	2317	39.2	3,506,807	48.9	269,675	42.7	40,486	42.5	26,103	39.0
	Male	64,407	53.1	33,333	55.6	3740	56.8	3598	60.8	3,670,350	51.1	362,414	57.3	54,784	57.5	40,788	61.0
Multiple gestation	Singleton	110,999	91.6	56,612	94.4	6204	94.3	5567	94.1	6,971,972	97.1	614,764	97.3	92,170	96.7	64,784	96.9
	Multiple	10,231	8.4	3333	5.6	376	5.7	348	5.9	205,197	2.9	17,327	2.7	3100	3.3	2107	3.1
Maternal age, y	<20	11,539	9.5	5997	10.0	749	11.4	694	11.7	688,641	9.6	73,439	11.6	12,043	12.6	8563	12.8
	20–24	25,552	21.1	13,253	22.1	1672	25.4	1452	24.5	1,588,727	22.1	156,782	24.8	25,264	26.5	18,221	27.2
	25–29	29,759	24.5	15,032	25.1	1671	25.4	1498	25.3	1,899,977	26.5	166,052	26.3	25,175	26.4	17,842	26.7
	30–34	29,250	24.1	14,298	23.9	1431	21.7	1271	21.5	1,771,955	24.7	141,320	22.4	19,939	20.9	13,615	20.4
	35–39	18,689	15.4	8538	14.2	779	11.8	745	12.6	981,925	13.7	75,607	12.0	10,300	10.8	6932	10.4
	40+	6436	5.3	2824	4.7	278	4.2	255	4.3	245,731	3.4	18,867	3.0	2545	2.7	1713	2.6
Nulliparity	Multiparity	73,852	60.9	37,599	62.7	4595	69.9	4025	68.1	4,369,555	60.9	396,376	62.7	66,100	69.4	46,168	69.0
	Nulliparity	47,331	39.1	22,326	37.3	1982	30.1	1888	31.9	2,805,715	39.1	235,548	37.3	29,140	30.6	20,703	31.0
Maternal race	Non-Latina White	33,204	28.7	15,518	27.1	1321	21.1	1182	20.7	1,985,227	28.9	147,085	24.3	21,505	23.6	12,339	19.1
	Non-Latina Black	8793	7.6	4184	7.3	480	7.7	470	8.2	383,794	5.6	36,622	6.0	5487	6.0	3899	6.0
	Non-Latina Asian	11,614	10.0	5591	9.8	431	6.9	431	7.6	798,010	11.6	59,355	9.8	6420	7.0	4325	6.7
	Non-Latina Pacific Islander	631	0.5	313	0.5	29	0.5	45	0.8	34,878	0.5	3631	0.6	570	0.6	466	0.7
	Latina	61,066	52.7	31,460	54.9	3975	63.5	3547	62.3	3,625,894	52.9	356,014	58.8	56,781	62.2	43,318	67.0
	Non-Latina AIAN	484	0.4	226	0.4	26	0.4	19	0.3	28,259	0.4	2686	0.4	441	0.5	299	0.5
	Non-Latina Other	70	0.1	34	0.1	4076	62.1	3	0.1	3771	0.1	310	0.1	38	0.0	31	0.0
Delivery payer	Medi-Cal	58,439	48.3	30,719	51.3	2298	35.0	3637	61.6	3,209,534	44.8	342,486	54.3	56,440	59.4	42,571	63.7
	Private	58,089	48.0	27,173	45.4	112	1.7	2053	34.8	3,673,591	51.3	268,389	42.5	35,652	37.5	22,090	33.1
	Self-pay/no charge/unattached	2339	1.9	1000	1.7	82	1.2	109	1.8	155,906	2.2	9774	1.5	1501	1.6	1005	1.5
	Other	2081	1.7	936	1.6	60	0.9	103	1.7	125,170	1.7	10,376	1.6	1491	1.6	1122	1.7
Prenatal care visits, n	0	1014	0.9	380	0.6	1581	24.7	44	0.8	31,126	0.4	2839	0.5	537	0.6	387	0.6
	1–9	28,960	24.4	12,691	21.6	4421	69.1	1522	26.4	1,216,103	17.3	114,141	18.5	18,091	19.4	12,600	19.4
	10–19	81,447	68.6	42,175	71.9	340	5.3	3928	68.1	5,509,151	78.2	474,126	76.7	70,421	75.7	49,463	76.0
	20+	7263	6.1	3385	5.8	-	0.0	274	4.8	289,797	4.1	26,830	4.3	4022	4.3	2650	4.1

AIAN, American Indian and Alaska Native; g, grams; n, number; RSV, respiratory syncytial virus; UB, unspecified bronchiolitis; wk, weeks; y, years.

## Discussion

We evaluated trends in admissions for RSV and other bronchiolitis for neonates born in California, stratified by risk status. RSV and UB hospitalizations are common in the first year of life. Consistent with previous reports, RSV-coded hospitalizations have declined over the past decade. This was true for the general population of infants, and the greatest declines were observed for infants with CLD, high-risk CHD, and early preterm birth. On the other hand, admissions for RSV in term infants without medical conditions and for UB across all infant groups remained relatively stable from 1998 to 2011.

Various interventions, including RSV immunoprophylaxis, may have contributed to the observed declines in RSV hospitalizations. In a study of patients with significant heart disease using a US national sample of pediatric hospital admissions, a 36% decrease in RSV hospitalizations was seen when comparing pre- and post-palivizumab guidelines for that population [9]. A similar finding was observed for high-risk CHD and CLD in a second study [1]. Although we did not have patient-level data on RSV immunoprophylaxis, other studies have demonstrated trends of increasing palivizumab use between 1998 and the 2004–2006 timeframe [11, 12]. These trends correspond to the temporal trend of declining RSV admissions from 1998 to 2006 seen in our results for infants with medical conditions and early gestational age. We speculate that infants with other high-risk conditions that have not been explicitly targeted for immunoprophylaxis may also have had increased usage of palivizumab due to recognition of their high-risk medical status for respiratory illness. Continuing to target high-risk groups with optimal treatment strategies and implementation of quality improvement should lead to sustained public health benefit.

Breastfeeding is considered a public health benefit in part due to its potential for decreasing infections in infancy [13]. National trends in breastfeeding increased after 1999 and extend beyond the end of our study period [14, 15]. Increases in breastfeeding may have contributed to declines in RSV hospitalizations during the study period. Presumably the increase in breastfeeding and the protection against infection that it confers would have been reflected in the number of UB hospitalizations; however, the decline was not as pronounced during the study period. Furthermore, one study indicated a significantly lower rate of exclusive breastfeeding in preterm infants (8% vs 19%) [16], and high-risk infants may be less likely to sustain breastfeeding due to their medical complexity.

We found that infants who were covered by Medicaid had higher rates of RSV and UB hospitalization (Table 2), a finding consistent with other studies [3]. These findings help validate this study’s analytic approach of using linked California datasets to evaluate infant hospitalization rates.

Although this and previous studies have shown declines in all infant RSV and UB hospitalizations between 1998 and 2012, the magnitude of the declines have differed across studies [1, 8]. In the current study, RSV and UB hospitalizations declined by approximately 38% and 25%, respectively, among infants without medical conditions, in the context of a 25% decline in infant hospitalizations due to any cause. Doucette et al found that RSV and UB hospitalizations decreased by 10% and 34%, respectively, among infants without medical conditions between 1997 and 2012, in the context of a 25% decline in all-cause infant hospitalizations [1]. Hasegawa et al demonstrated a 21% reduction in infant bronchiolitis hospitalizations due to RSV and other causes between 2000 and 2009 [8]. The larger decline in RSV hospitalizations in the current study may be due to decreased testing for RSV during the study interval in California relative to other parts of the country, perhaps due to an increased prevalence of managed care and adoption of recommendations against routine testing for RSV, which would in turn lead to a decrease in RSV-coded respiratory illnesses [17].

Although there was a general decline in RSV hospitalizations during the study period, there was a sudden spike in RSV hospitalization rates in 2010 for most groups (Figs 2C and 3A), with a spike also seen among infants born in 2009 at <28 weeks GA. However, by 2011, hospitalization rates were again consistent with the general trajectory observed in previous years. It is unclear why this spike in 2010 occurred. One speculation is that this was due to the timing of the 2009–2010 influenza pandemic, which likely inhibited RSV circulation and increased concern and confirmatory testing for viral respiratory disease, leading to increased incidence and diagnosis of RSV hospitalizations among at-risk groups the following RSV season. Additional factors such as seasonal differences in RSV strain pathogenicity could have also played a role. As additional years’ data become available, it will be important to evaluate if 2010 is an anomaly or part of a trend of increasing annual variability. If the spike was due to increased testing during the 2009–2010 pandemic, the disease rates in 2010 can provide some insight into what RSV and UB hospitalization rates might be if there was more comprehensive laboratory testing for RSV.

A limitation of this study that should be considered is that this was an analysis of California hospitalizations of infants born in the state, and admissions could not be captured when infants moved out of state or had hospitalizations outside of California. It should also be noted that given the sample size, even small differences were expected to be statistically significant; as a result, the primary presentation of the results focuses on clinically meaningful differences rather than on statistical significance.

In summary, we found a general decline in RSV hospitalizations across a large population-based cohort. This was particularly true for high-risk conditions that are recommended for RSV immunoprophylaxis, such as CLD, high-risk CHD, and preterm birth at early GA. During this study period, the level of hospitalizations for UB remained relatively stable. As there appear to be interventions and preventive efforts that may alter outcomes, it will be important to continue research into which populations will benefit from targeted interventions and implement appropriate measures to provide maximal public health benefit.

## Supporting information

S1 Table Medical conditions for categorization. ^a^Infants with these conditions were excluded from analyses of risk by gestational age among infants without serious medical conditions.
(DOCX)
