# Supplementary material for: Population-based trends and underlying risk factors for infant respiratory syncytial virus and bronchiolitis hospitalizations
Source: PLoS One. 2018 Oct 31;13(10):e0205399. doi: 10.1371/journal.pone.0205399 (PMC6209180; doi:10.1371/journal.pone.0205399)
Supplement: S1 Table — aInfants with these conditions were excluded from analyses of risk by gestational age among infants without serious medical conditions. (DOCX) [file pone.0205399.s001.docx]

**Supporting Information**

**S1 Table. Medical Conditions for Categorization**

| **Condition** | **ICD-9 Codes** |
| --- | --- |
| ^a^Chronic respiratory disease arising in the perinatal period (ie, chronic lung disease) | 770.7X |
| ^a^Congenital anomalies of the respiratory system | 748.XX |
| ^a^Higher-risk congenital heart disease | 425.4X; 428.0X; 745.0X- 745.4X; 745.6X – 745.8; 746.01– 746.5X; 746.7X – 746.85; 746.87; 747.1X; 747.21 – 747.49 |
| Lower-risk congenital heart disease | 745.5X; 745.9X; 746.00; 746.6X; 746.86; 746.89 – 746.9X; 747.0X; 747.20; 747.83 |
| Down syndrome | 758.0X |
| ^a^Cystic fibrosis with pulmonary manifestations | 277.0X |
| ^a^Neuromuscular disease | 330.XX; 335.XX; 343.XX; 356.XX; 358.1; 359.0X – 359.23; 359.9; 775.2 |
| ^a^Human immunodeficiency virus | 079.51, 079.52, 079.53 |
| ^a^Immunodeficiency | 279.XX |
| ^a^Congenital and metabolic | 740.XX; 741.XX; 742.XX; 754.2; 756.1X; 756.6; 758.1 – 758.9; 759.3; 759.7X – 759.9X; 271.0; 272.7; 277.5; 277.81; 277.82; 277.86 |

^a^Infants with these conditions were excluded from analyses of risk by gestational age among infants without serious medical conditions.
